# Mesoporous nanodrug delivery system: a powerful tool for a new paradigm of remodeling of the tumor microenvironment

**DOI:** 10.1186/s12951-023-01841-2

**Published:** 2023-03-21

**Authors:** Yinhui Hang, Yanfang Liu, Zhaogang Teng, Xiongfeng Cao, Haitao Zhu

**Affiliations:** 1 Department of Medical Imaging, Affiliated Hospital of Jiangsu University, Zhenjiang, 212001 People’s Republic of China; 2 Institute of Medical Imaging and Artificial Intelligence, Jiangsu University, Zhenjiang, 212001 People’s Republic of China; 3 Laboratory of Medical Imaging, The First People’s Hospital of Zhenjiang, Zhenjiang, 212001 People’s Republic of China; 4 Key Laboratory for Organic Electronics and Information Displays & Jiangsu Key Laboratory for Biosensors, Institute of Advanced Materials (IAM), Nanjing University of Posts and Telecommunications, Nanjing, 210023 People’s Republic of China

**Keywords:** Tumor microenvironment, Metabolism, Mesoporous nanodrug delivery systems, Targeted remodeling, Tumor therapy

## Abstract

Tumor microenvironment (TME) plays an important role in tumor progression, metastasis and therapy resistance. Remodeling the TME has recently been deemed an attractive tumor therapeutic strategy. Due to its complexity and heterogeneity, remodeling the TME still faces great challenges. With the great advantage of drug loading ability, tumor accumulation, multifactor controllability, and persistent guest molecule release ability, mesoporous nanodrug delivery systems (MNDDSs) have been widely used as effective antitumor drug delivery tools as well as remolding TME. This review summarizes the components and characteristics of the TME, as well as the crosstalk between the TME and cancer cells and focuses on the important role of drug delivery strategies based on MNDDSs in targeted remodeling TME metabolic and synergistic anticancer therapy.

**Graphical Abstract:**

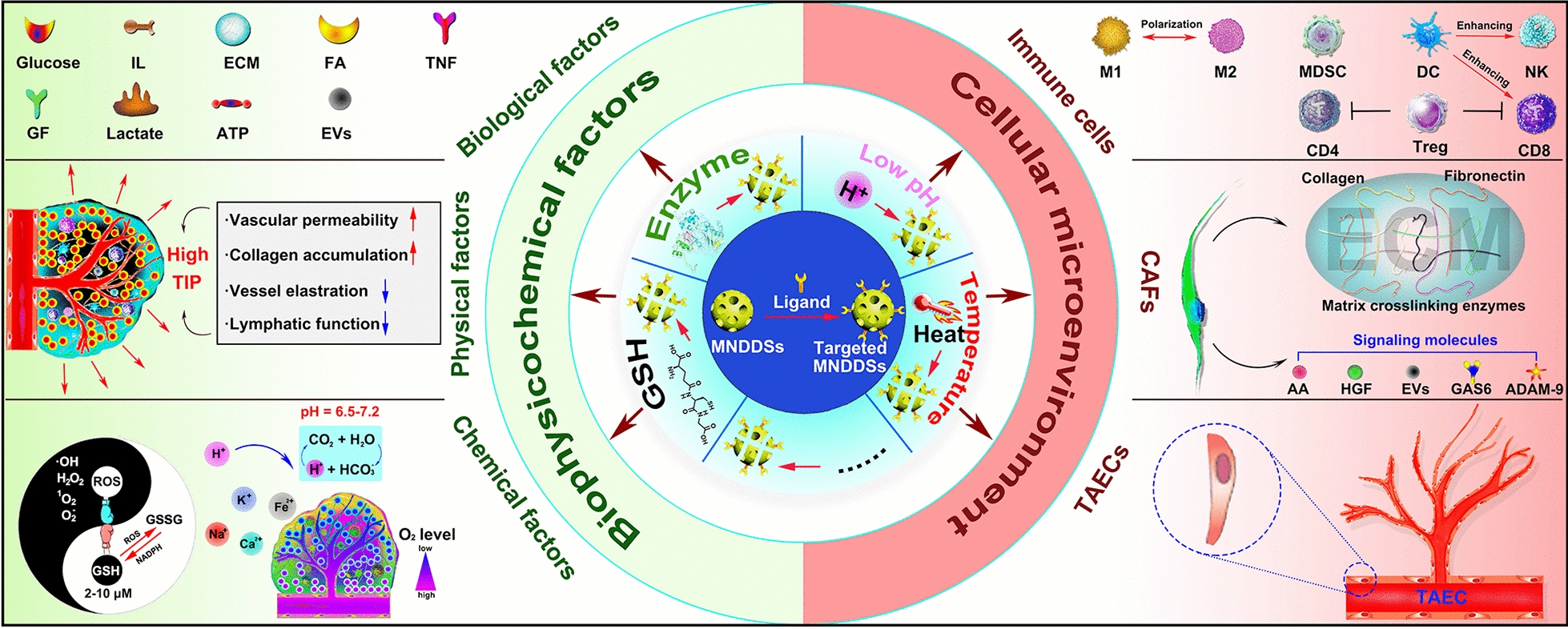

## Introduction

Tumor microenvironment (TME), as a complex environment, can be roughly divided into the cellular TME and the biophysiochemical TME. The cellular TME consists mainly of all noncancer cells, such as cancer-associated fibroblasts (CAFs), endothelial cells, and immune cells. The biophysiochemical TME includes the extracellular matrix, metabolites, signaling molecules and soluble products, small extracellular vesicles, oxygen partial pressure and interstitial pressure, pH, etc. The TME, as the “soil”, provides a suitable environment for the growth of cancer cells and plays an important role in the occurrence, progression, metastasis, recurrence and treatment resistance of tumors [1]. Due to the crucial role of the TME in tumor survival and treatment resistance, remodeling the TME may contribute to the cure of cancers, which is currently a hot topic in cancer therapy.

Emerging mesoporous nanodrug delivery systems (MNDDSs) have been facilitated as a novel therapeutic approach for remodeling the TME. As an advanced nanodelivery system, mesoporous nanoparticles significantly enhanced drug loading efficiency and realized all kinds of chemotherapy drugs safe, precise and efficient delivery to the tumor site [2, 3]. Moreover, modified MNDDSs can recognize and target both cancer cells and reshape the TME.

In this review, the TME regulation of cancer cell biology and targeted remodeling of the TME by MNDDSs are reviewed. We first discussed the composition and characteristics of the TME and then reviewed the interaction between the TME and cancer cells, especially focusing on the TME promoting tumor survival and therapeutic resistance. Finally, the advantages of the MNDDSs for targeting and reshaping the TME were introduced, and the biggest remaining challenges in this field were summarized.

## The tumor microenvironment

### Composition and characteristics of the TME

**Fig. 1:**
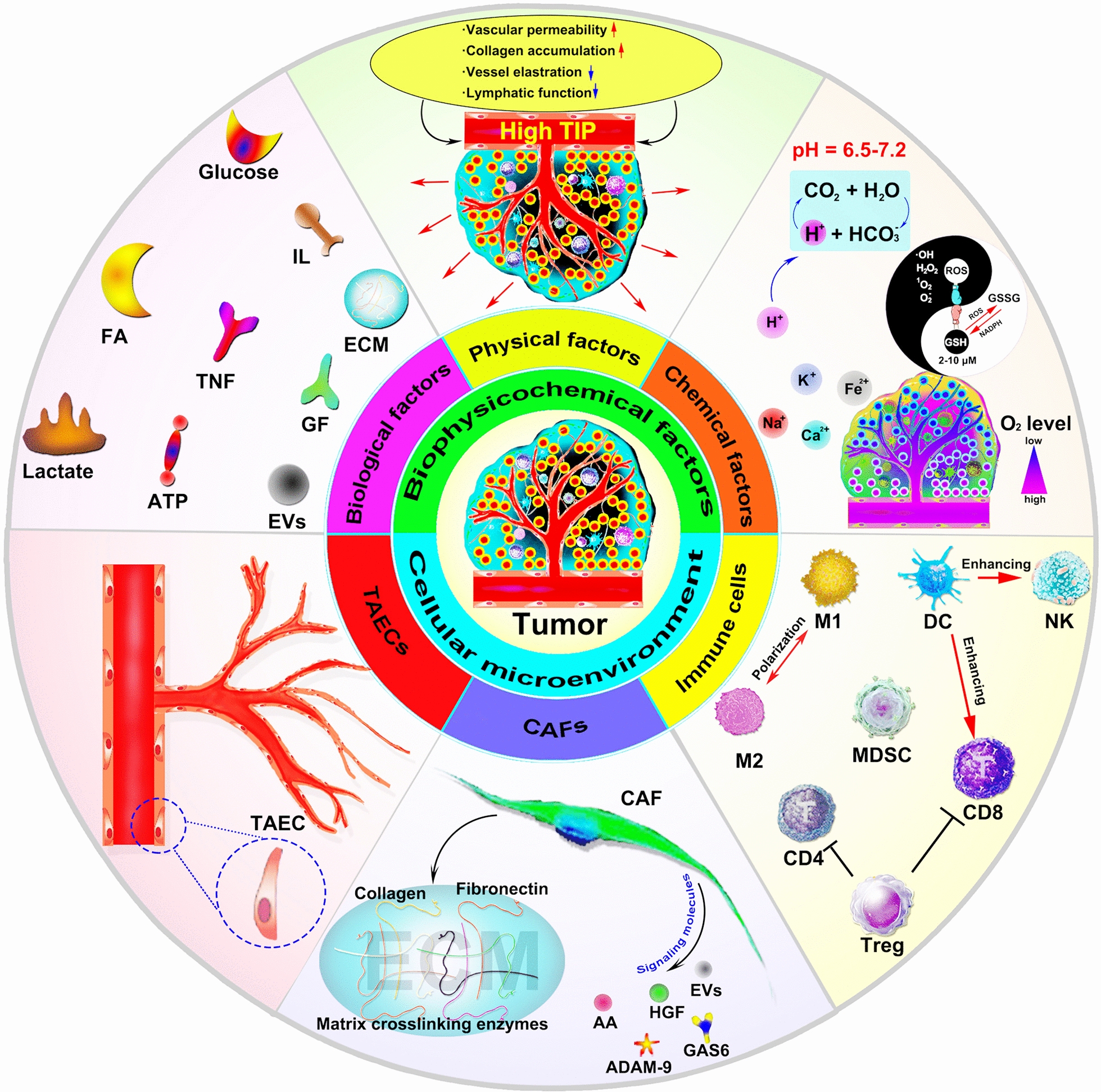
Schematic presentation of the TME. Various cell types and non-cell components are involved to support tumor proliferation, invasion, and metastasis

The TME can be roughly classified into two categories: the cellular TME and the noncellular TME. The cellular TME consists of vascular endothelial cells, fibroblasts, immune cells, etc. The noncellular TME refers to the extracellular matrix (ECM) surrounding cancer cells and stromal cells, which can be divided into three categories: (1) biological factors: energy materials (glucose, amino acids, fatty acids, lactate), cytokines (interleukin, interferons, tumor necrosis factor superfamily, colony stimulating factor, chemokines and growth factors), ECM (collagen, elastin, proteoglycan and amino chitosan), etc.; (2) physical factors: interstitial pressure; (3) chemical factors: pH, oxygen, carbon dioxide, nitric oxide (NO), ions (K^+^, Na^+^, Ca^2+^, Fe^2+^, etc.), etc. (Fig. 1).

### Cellular microenvironment and its biology

Cancer cell biology is regulated by both intrinsic factors and the surrounding stromal cells, such as endothelial cells, fibroblasts and immune cells.

#### Tumor neovascularization

Tumor neovascularization functions by delivering various nutrients and oxygen to the tumor and removing metabolic waste. In addition, the number, maturity and distance of tumor vessels play key roles in tumor therapy efficacy [4]. The distinct prognosis of early and advanced renal cancer may be ascribed to the neovascularization density, vascular endothelial cell size, and proliferation capacity [5]. Moreover, microvessel density is a meaningful prognostic factor in non-small cell lung cancer (NSCLC), colorectal cancer, and breast cancer [6–8].

Tumor neovascularization results from tumor-associated endothelial cells (TAECs) and forms dysfunctional capillaries of blood vessels, which are induced by noncellular microenvironments. The direct crosstalk between TAECs and cancer cells may contribute to the process of tumor angiogenesis. The mitogen-activated protein kinase (MAPK) and Notch signaling pathways are thought to be critical factors. The Notch signaling pathway is involved in the differentiation of apical cells, proliferation of endothelial cells, and formation of mature vascular structures [9]. In addition, e-selectin and ligand sialyl Lewis oligosaccharide X are also involved in tumor neovascularization [10]. In addition, the hypoxic and acidic microenvironment could induce and stabilize HIF1-α expression, upregulates vascular endothelial growth factor (VEGF) expression levels, and promotes tumor neovascularization [11, 12]. Moreover, VEGF can also induce cancer cells to shift from oxidative phosphorylation (OXPHOS) to glycolysis, indirectly leading to lactate accumulation in the microenvironment, forming a positive feedback effect [13, 14].

#### Cancer-associated fibroblasts

As one of the most important stromal cells in solid tumor, CAFs may transform into various subtypes of CAFs under the stimulation of stress, inflammation and cancer cells and express α-smooth muscle actin (α-SMA) and fibroblast activation protein (FAP). CAFs could mediate cancer cell growth, migration and invasion depending on secreting various nutrients (lactate, glutamine) [15–17] and signaling molecules, such as aspartic acid, hepatocyte growth factor (HGF), VEGF, growth arrest specific protein 6 (GAS6), and exosomes [18–21]. CAF-derived ADAM-9 is positively associated with melanoma cell proliferation, apoptosis resistance, migration and invasion [22]. In addition, CAFs derived type I, III, and V collagen and fibronectin and matrix crosslinking enzymes can remodel the ECM and provide a supporting skeleton for cancer cell survival and proliferation [23].

CAFs also contribute to cancer cell therapy resistance and recurrence [24]. CD10^+^ GPR77^+^ CAF subtypes may provide a survival niche for cancer stem cells (CSCs), which are thought to be the seed of chemoresistance and recurrence [25]. In addition, CAF-derived soluble factors and cell adhesion molecules could activate the cancer cell antiapoptotic response and drug resistance-related signaling molecules. CAF-derived HGF could increase receptor tyrosine kinase (RTK) ligand level in melanoma cells, further activating the downstream effector factors phosphatidylinositol-3-OH kinase (PI3K) and MAPK and thus promoting resistance to kinase inhibitors [26]. CAF-secreted interleukin (IL) -6 activates the transformation of epithelial-mesenchymal phenotypes of esophageal adenocarcinoma cancer cells, which further enhances therapy resistance, migration, and clonogenesis of cancer cells [27].

In addition, CAFs are also associated with an immunosuppressive TME and cancer cell immune escape. CAFs secreted collagen fibers that increase the viscosity of the ECM and impede immune cell infiltration, which ultimately results in T-cell depletion and tumor immune tolerance [28]. CAF-secreted chemokines, such as IL-6, C–X–C motif chemokine ligand 9 (CXCL9) and TGF-β, can regulate the migration of infiltrating leukocytes [29]. Moreover, CAFs can also inhibit natural killer (NK) cell functions depending on Netrin G1 [30]. KRAS-mutant pancreatic cancer cells can enhance myeloid-derived suppressor cell (MDSC) infiltration into tumors and lead to anti programmed cell death 1 immunotherapy resistance [31].

#### Immune cells

Tumor-infiltrating immune cells are a complex society, including innate immune cell subpopulations, such as NK cells, macrophages and dendritic cells (DCs), and adaptive immune cell subpopulations, such as CD8^+^ T and CD4^+^ T cells.

Tumor immune escape results mainly from the dysfunction of cytotoxic T cells (CTLs) [32]. Inhibitory receptors, such as programmed cell death 1 (PD-1), lymphocyte activation gene-3 (LAG-3), T-cell immunoglobulin-3 (TIM-3), and cytotoxic T-lymphocyte-associated protein 4 (CTLA-4), are highly expressed in tumor-infiltrated CD8^+^ T cells, which results in CD8^+^ T-cell exhaustion [33, 34].

Tumor-associated macrophages (TAMs) can polarize into either M1-like macrophages with proinflammatory (antitumor) function or M2-like macrophages with anti-inflammatory (protumor) function [35]. M2-TAMs are rich in tumor tissues, promote vascular production and degrade ECM, provide nutrition for tumor growth, and promote tumor development and metastasis [36]. Lactate can promote the transformation of the macrophage phenotype from M1 to M2, while glutamate accumulation can induce the reverse process [37, 38].

DCs are the most powerful antigen-presenting cells in the body, with strong antigen uptake and processing capabilities, and can present tumor antigens to primary T cells [39]. DCs trigger specific immune responses against cancer cells by enhancing the function of CD8^+^ T cells or NK cells [40, 41]. Inhibitory cytokines in the TME may lead to DC dysfunction, which results in cancer cells escaping the surveillance of the immune system [42, 43]. NK cells can directly kill cancer cells and promote adaptive immunity by secreting cytokines, playing a crucial role in the antitumor process [44]. However, cancer cells can induce dysfunction of NK cells and evade the surveillance of NK cells [45, 46]. As classic immune suppression cells, infiltrated Tregs in tumors can secrete immunosuppressive factors, directly killing or inhibiting the proliferation of effector T cells [47].

### Biophysiochemical microenvironment and its biology

The biophysiochemical microenvironment is a critical connection between cancer cells and the cellular microenvironment, also plays a crucial role in cancer cell biology.

#### Biological factors

Biological factors include metabolism materials (glucose, amino acids, fatty acid, and lactate), cytokines (IL, interferons (IFNs), tumor necrosis factor superfamily, colony stimulating factor, chemokines and growth factors), ECM (collagen, elastin, proteoglycan and amino chitosan) and small extracellular vesicles.

TME metabolites are the main energy source of cancer cells and play an important role in tumorigenesis, recurrence and metastasis. Cancer cells obtain energy depending on glycolysis derived lactate at the cost of consuming glucose, called aerobic glycolysis or Warburg effect [48]. Sotgia et al. suggested that the stromal cells derived metabolites (L-lactate and ketone bodies) could be transport into epithelial cancer cells, and further drive OXPHOS and mitochondrial metabolism, which was termed the “reverse Warburg effect” [49]. Both glucose metabolism pattens are essential for tumor biology. Lactate is either one of the critical cancer cell energy materials or biological factors in the TME. Maria et al. found that lactate in the TME contributed to cancer cell chemotherapy resistance [50]. ECM components, especially collagens, also contribute to drug resistance. Collagen fibers form a dense physical barrier that blocks pancreatic cancer cell from taking up chemotherapy agents [51]. Similar phenomena can be detected in breast and colon cancer cell models [52].

Chemokines participate in TME remodeling and tumor progression [53]. Chemokines (IL1, 6, 12, and 23) remodel the immune ECM, promote the expression of iNOS, and ultimately promote tumor progression [54]. Chemokine ligand 2 (CCL2) expression is positively correlated with poor prognosis in breast and bladder cancer [55, 56]. Moreover, the feedback between chemokines and cancer cells makes tumors “unhealed wounds”.

Extracellular vehicles (EVs), including exosomes, microvesicles, and large oncosomes, are involved in the transmission of signals or other molecules between cancer cells and stomal cells [57]. As a key component of the TME, EVs play different roles in tumor immunity escape, proliferation, metastasis and therapy sensitivity. Therefore, a deeper and more comprehensive understanding of how EVs integrate between cancer cells and the TME may represent a novel cancer treatment strategy.

#### Physical factors

High interstitial pressure is the characteristic of solid tumor microenvironment, which contributes to tumor progression and therapy resistance [58]. High tumor interstitial pressure results mainly from internal and external factors. The internal factors include: (1) a high concentration of collagen accumulation; (2) high tumor vascular permeability; (3) poor elastration performance and vulnerable vessel walls; and (4) a dysfunctional lymphatic system [59]. External factors refer to the external pressure on the tumor [60]. Increased interstitial pressure limits the continuous perfusion of blood to the tumor site, which results in chemotherapy drugs, monoclonal antibodies and immune cells hardly accumulating in the tumor [61]. Pancreatic ductal adenocarcinoma (PDAC) TME is characterized by excessive fibrosis and extracellular matrix deposition, resulting in high interstitial pressure, vascular collapse, and low diffusion of nutrients and oxygen. Chemotherapy drugs and immune cells also hardly penetrate into the tumor, leading to treatment resistance and immunity escape [62]. Moreover, the tortuosity, leakage of the vessel walls and dysfunctional lymphatic system will lead to uneven blood flow and local fluid accumulation, increase the interstitial pressure, which further obstacles chemotherapy agents or macromolecule transport into the TME [63, 64]. Therefore, reducing the interstitial pressure may improve chemotherapy effective. Thus, targeting ECM components, anti-angiogenesis, normalizing blood vessels, physical operation or combination of all these factors may bring new inspiration to the solid tumor treatment.

#### Chemical factors

Chemical factors include low pH, oxygen, glutathione (GSH), reactive oxygen species (ROS), carbon dioxide, NO, ions (K^+^, Na^+^, Ca^2+^, Fe^2+^, etc.), etc. Hypoxia is considered to be one of the most important factors in the TME, resulting from unlimited proliferation of cancer cells and dysfunctional blood vessels [65]. Hypoxia is closely associated with poor clinical prognosis, increased genomic instability, increased chemotherapy or radiotherapy resistance, immunosuppression, CSCs enrichment, and metastasis. Mainly, hypoxia induces activation of hypoxia inducible factor 1-alpha (HIF-1α) and its downstream genes related to cell metabolism, survival, movement, basal membrane integrity, angiogenesis, and hematopoiesis, which promotes cancer cell proliferation, invasion and metastasis [66–68]. In addition, hypoxia could also regulate overall mRNA homeostasis and enhance stress tolerance [69].

Low pH is another feature of the TME, which results mainly from metabolic materials and a variety of ion effluxes [70]. Low pH contributes to cancer cell apoptosis resistance, proliferation, and multiple drug resistance (MDR) [71]. Vacuolar proton pumps (V-ATPases), which function to pump H^+^ to the extracellular space or intermembrane [72], can maintain the neutral cytoplasm and extracellular acidic environment and avoid self-acidosis. Moreover, the accumulation of H^+^ around cancer cells can activate the enzyme cascade reaction and induce proteolytic enzyme secretion, which contributes to the degradation or reconstruction of the ECM and tumor invasion and metastasis [73].

High concentration of GSH is another characteristic of the TME [74]. GSH could protect cancer cells from both oxidative stress damage and the toxicity of exogenous electrophiles, maintaining redox homeostasis. ROS could both directly oxidizes GSH to GSSG, and reaction with niacinamide adenine dinucleotide phosphate (NADPH) to form GSH. Also, GSH could act as a cofactor to reduce hydroperoxide substrates [75]. Hence, overexpressed GSH in the TME would seriously scavenge ROS, weakening the radiotherapy, chemotherapy and chemodynamic therapy (CDT) efficiency. Reducing GSH levels in the TME has become a potential target for cancer treatment [76].

## The mesoporous nanodrug delivery systems target the TME

### Classification of mesoporous nanodrug delivery systems

**Fig. 2:**
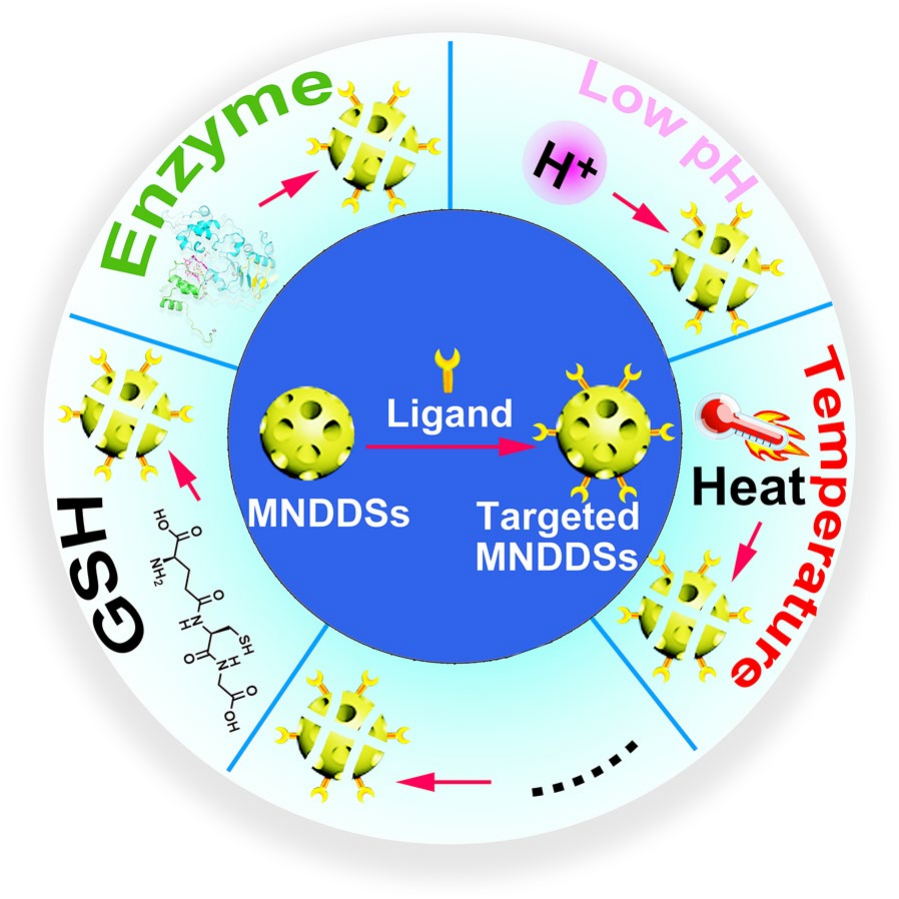
Multifunctional modified MNDDSs respond to stimulus in vitro and in vivo to target remodeling tumor microenvironment

MNDDSs are currently hotspots in tumor diagnosis, monitoring and treatment. MNDDSs are nanodrugs synthesized based on porous nanomaterials with a diameter of 2–50 nm. According to the chemical component of the nanosystem, MNDDSs can be divided into inorganic structures (such as oxide, mesoporous carbon, mesoporous nitrogen, phosphate, sulfides and monoatomic mesoporous materials), organic structures (such as polymer, mesoporous organosilicon, etc.) and inorganic–organic hybrid structures (such as metal-organic frameworks) [77]. Also, based on the spatial distribution characteristics, MNDDSs can be divided into ordered (regular pore arrangement) and disordered (irregular pore size distribution). According to the pore structure, MNDDSs can be divided into hexagonal (MCM41), cubic (MCM48) and layered mesoporous (MCM50), etc. According to the shape, it can be divided into mesoporous particles, mesoporous membranes and mesoporous three-dimensional bodies. According to the TME response, MNDDSs can be divided into pH-sensitive, enzyme-sensitive, temperature-sensitive, reduction-sensitive and photosensitive delivery systems (Fig. 2) [78]. In our review, MNDDSs can be roughly divided into cellular TME targeted (TAEC targeted, CAF targeted, immune cell targeted, etc.) and biophysiochemical TME remodeled (pH, energy metabolism, redox homoeostasis, hypoxia remodeled, etc.)

### Properties and advantages of MNDDSs in the TME

MNDDSs with the following advantages: (1) intrinsic physical advantages of mesoporous nano-systems, including high loading capability, controllable pore size, morphology and framework control; (2) highly multifunctional modification ability as the drug delivery systems [79].

#### Intrinsic physical advantages

The multi-porous structure of mesoporous materials provides huge space for the cargo transportation and ensures high drug loading. In particular, the hollow mesoporous nanomaterials have expanded the internal space. Zhang et al. developed the intelligent triple-PS_S_ (mesoporous carbon nitride, nitrogen-doped graphene quantum nitride and photofrin) hybrid nano-regulator could simultaneously respond to UV–vis light, generate higher rate of ROS, and improve the therapeutic effects [80]. The high load ability of MNDDSs ensures a large number of cargoes, which provides a prerequisite for the efficient performance of nanodynamic therapy. In addition, the controllable aperture is more suitable for carrying different types of cargoes, such as drugs, protease, nucleic acid, and ultra-small NPs [81–83].

The shape of MNDDSs would affect their movement mechanism in the circulation or pass through the biological barrier, performing higher rate of cell uptake and tumor inhibition [84, 85]. Therefore, to improve their behavior, virous shapes of MNDDSs were constructed, such as nanospheres [86], core–shell structures [87], dendritic and tubular structures [88, 89]. Huang et al. revealed that different forms of mesoporous silica nanoparticle (MSN) function distinct in human melanoma A375 cells [90]. Moreover, to diversify the function, additional elements were integrated into the skeleton design of MNDDSs, such as inorganic metal ions [91], organic macromolecules [92], organic functional groups [93], etc. Compared with purely inorganic skeleton, framework control enhances the optimization of MNDDSs therapeutics.

#### Multifunctional modification

Surface modification also plays an irreplaceable role in the functional diversification of MNDDSs. MNDDSs encapsulated with biological membranes (red cell membrane, cancer cell membrane) have better biocompatibility and achieved high accumulation in target tissues [94, 95]. Integrating polyethylene glycol biocompatible polymers into mesoporous materials reduced the clearance of blood proteins and macrophages, and prolong the blood circulation time of MNDDSs [96]. Taking advantage of the specific expression characteristics of some receptors in tumor cells, mesoporous materials such as folic acid (FA) [97], hyaluronic acid (HA) [98], glucose protein 78 peptides (GRP78P) [99, 100] can be modified with ligands to achieve specific targeting purposes. Similarly, the biochemical characteristics of TME, such as pH, GSH and hypoxia, could act as the activator for the gatekeeper response and drug release of MNDDSs [78, 101]. These specific-targeted designs enable more precise drug delivery and tumor localization, more comprehensive diagnosis and treatment of local and systemic lesions. Finally, the modification of nano sensitizers can make MNDDSs triggered by exogenous/endogenous activators or internal chemical/biological reactions in the TME, which can be used for image-guided phototherapy, thermology and dynamic therapy [102, 103]. The diversified functional modification on the surface of MNDDSs allow its wide application in nano-dynamic therapy, providing an excellent reference for future design.

## MNDDSs reshape the cellular microenvironment

### Remodeling the blood vessel system

**Fig. 3:**
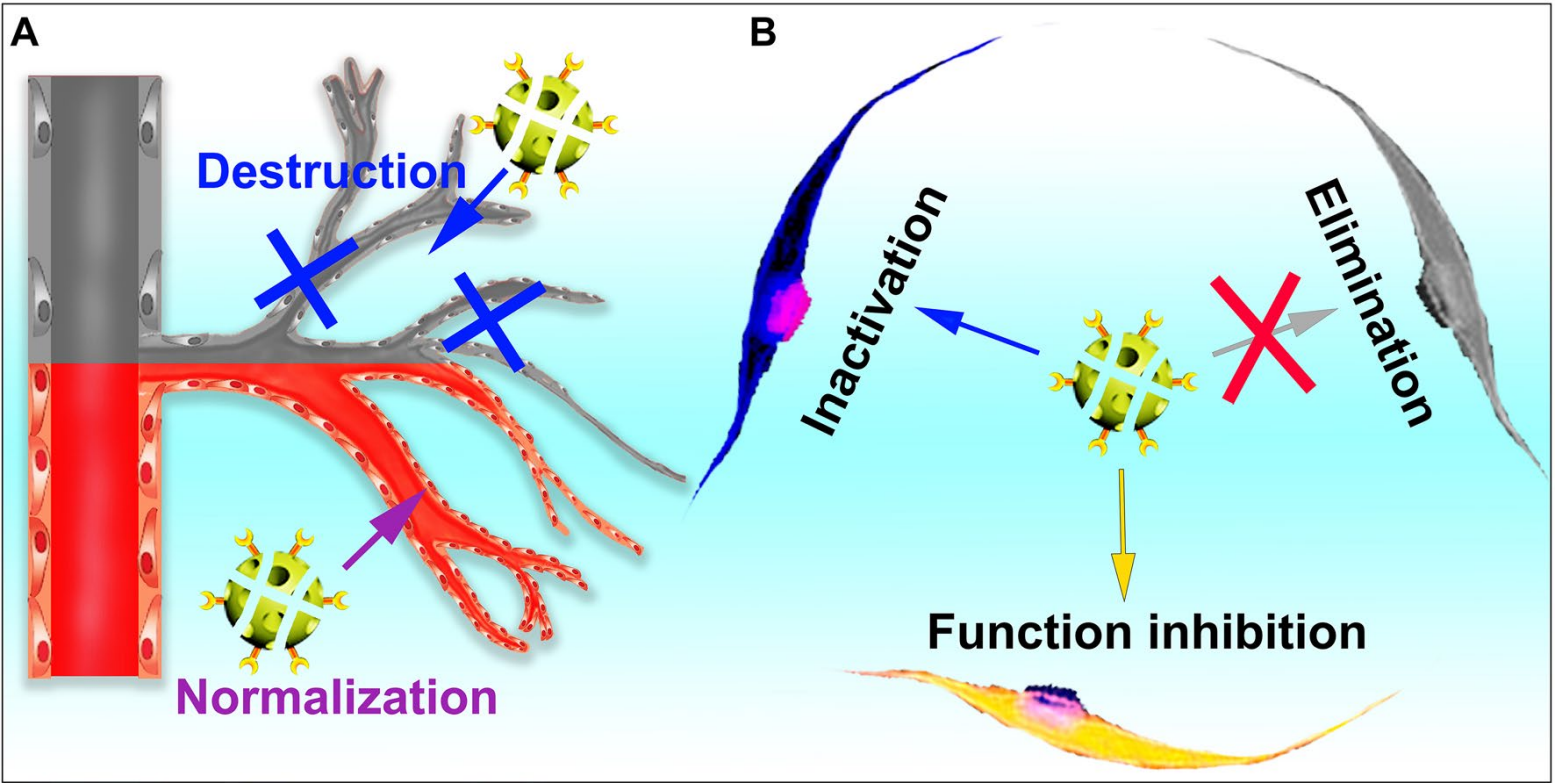
**A** Strategies for remodeling tumor blood vessel systems currently include blocking neovascularization formation, destructing existing blood vessels and normalizing tumor vessels. **B** MNDDSs could eliminate CAFs, inhibit the function of CAFs, and reverse CAFs to a quiescent condition or a tumor suppressor phenotype

Tumor vascular therapy strategies currently include tumor angiogenesis inhibitors and promoting tumor vascular maturation (Fig. 3).

#### Blocking neovascularization formation

Tumor angiogenesis inhibitors may directly or indirectly antagonize angiogenic factors. Direct vascular inhibitors, such as bevacizumab (Avastin), Endostar, and siRNA VEGF, can inhibit the expression and function of proangiogenic factors [104, 105]. Hu et al. constructed a cervical cancer-targeted gold nanorod-MSN for the codelivery of cisplatin and the antiangiogenic drug Avastin to achieve efficient vascular inhibition [106]. Chen et al. built a magnetic MSN carrier, delivering siRNA VEGF to silence VEGF in the tumor tissue and inhibit angiogenesis [107]. Recently, several studies also revealed that VEGF inhibition may also contribute to the immunosuppressive TME [108]. Combining antiangiogenic agents and immune therapy could significantly improve tumor inhibition. Moreover, the application of mesoporous nanomaterials as a drug delivery system is thought to be a promising strategy for this combination therapy.

Tyrosine kinase inhibitors (TKIs), such as sorafenib (SO), nintedanib, lenvatinib, and axitinib, can simultaneously target multiple pathways associated with VEGF, PDGFR and FGFR, which are indirect multitarget angiogenesis inhibitors in the future [109–111]. Zhao et al. developed pH-sensitive MSNs USMNS-Cl, which were used for the controlled release of SO and ursolic acid (UA) [112]. Compared with UA alone, USMNS-Cl significantly downregulated the expression of epidermal growth factor receptor (EGFR) and vascular endothelial growth factor receptor 2 (VEGFR2) and inhibited tumor angiogenesis in vitro and in vivo. Due to the role of lactate in promoting tumor neovascularization [113], consuming lactate may represent a good choice to antagonize the formation of abnormal tumor blood vessels to a certain extent. Tang et al. used dendritic MSN (ODMSN)-loaded lactate oxidase (LOX) as a carrier to antagonize tumor angiogenesis by consuming 99.9% TME lactate, downregulating VEGR and inhibiting angiogenesis [114].

#### Destruction of existing blood vessels

The destruction of the existing blood vessels may result in cancer cell starvation and death [115]. Liu et al. constructed a hollow mesoporous silica nanodrug delivery system (tHMSN) modified with TLYP-1 and doxorubicin (DOX), which displayed strong cytotoxic effects for breast cancer cells and tumor umbilical vein endothelial cells [116]. Veeranarayanan et al. synthesized a dual-drug and DNA fluorescence dye DAPI-loaded monodisperse mesoporous silica microsphere (MSN-FT), which could specifically target and completely disrupt cancer cell migration and angiogenic germination of activated endothelial cells [117]. Although targeting to destroy tumor blood vessels was achieved, targeting to destroy tumor blood vessels also prevented antitumor drugs from reaching core tumor tissue, which may result in therapy resistance, recurrence, and metastases [118].

#### Normalize tumor vessels

The imbalance between proangiogenic factors and antiangiogenic factors leads to vascular abnormalities [119]. Normalized tumor blood vessels have attracted great attention recently and can decrease interstitial pressure, increase oxygen content, improve an immunosuppressive TME, deliver drugs into the tumor tissue, and enhance therapeutic effects [120, 121]. Dopamine-loaded nanoparticles (NPs@DA) could release dopamine in a weakly acidic environment, which further significantly inhibited the migration of vascular endothelial cells and the formation of tubules, induced the normalization of tumor vessels, and thus improved the antitumor chemotherapy effect of DOX. Moreover, NPs@DA also upregulated Ang1 in pericytes and Kruppel-like factor-2 (KLF2) in endothelial cells and inhibited VEGF expression [122]. Although normalization of tumor vessels is another promising strategy, there are few reports using MNDDSs in this area.

### Reshape the CAFs microenvironment

With the enhanced permeability and retention (EPR) effect and special physical characteristics, modified MNDDSs could eliminate, inactivate or inhibit the function of CAFs and further break CAFs-formed physical barriers, which ultimately enhanced drug delivery and tumor inhibition efficacy.

As losartan (LOS) could inhibit CAFs from secreting collagens, Zhang et al. encapsulated LOS in hollow mesoporous Prussian blue nanoparticles (HMPBs) and constructed the (LOS + DOX) @HMPBS platform, which realized ECM degradation, improved the penetration ability of DOX in tumors and inhibited tumor growth [123]. Hou et al. also constructed a CAF-targeted molecule AEAA-modified Pep-APCDs@Fe/DOX-LOS mesoporous carbon nanodot platform for the targeted delivery of LOS, DOX, and Fe ions. This platform inhibited CAF function and enhanced the deep tumor penetration of DOX and Fe ions in tumor tissues and therapeutic efficiency [124].

Elimination of CAFs can enhance the penetration of therapeutic drugs into the tumor, improve the immunosuppressive microenvironment and enhance the antitumor immune response [125, 126]. Elimination of CAFs is usually accompanied by serious side effects, such as increasing the risk of tumor metastasis and invasion, promoting epithelial to mesenchymal transition, and triggering chemotherapy resistance [127].

Reversing CAFs to a quiescent condition or a tumor suppressor phenotype is currently a hot topic, and this objective may be precision-realized using the MNDDSs. Additionally, MNDDSs used to impede CAFs to secrete chemokines have not been reported and deserve deeper investigation in the future [128].

### Remodeling the immune microenvironment

**Fig. 4:**
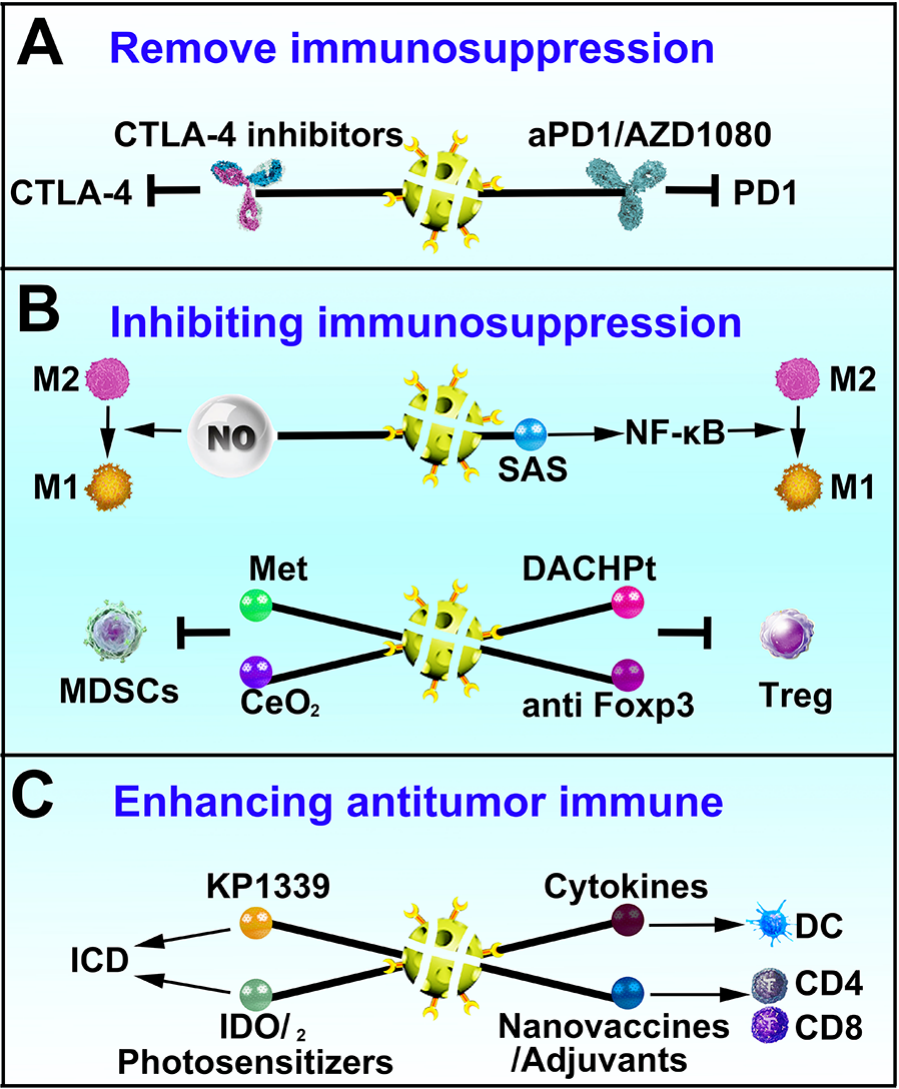
Strategies for remodeling immune environment. **A** Remove immunosuppressive factors, such as PD-1 and CTLA-4, etc. **B** Inhibit function of immunosuppressive subpopulation, such as TAMs (M2 phenotype), MDSCs and Tregs. **C** Enhance antitumor immune in the form of inducing cancer cells immunogenic cell death (ICD), damage-associated molecular patterns (DAMPs) or antigen release; delivering adjuvants or cytokines, and co-stimulating activation of immune cell

Due to the immunosuppressive state of the TME and exhausted immune cells, remodeling the immune microenvironment is of great significance for cancer treatment (Fig. 4).

#### Removal of immunosuppressive factors

PD-1 and CTLA-4 are the most common T-cell functional inhibitors [129]. Zhao et al. designed pH-responsive cancer cell membrane-camouflaged MSNs (DTIC@CMSN) to deliver dacarbazine (DTIC) and PD-1 antibody (aPD1) to achieve superior antitumor effects [130]. DTIC@CMSN + aPD1 activates tumor-specific T cells and reverses the immunosuppressive TME. In colorectal tumor, pancreatic, and lung tumor models, Allen et al. demonstrated that the GSK3 inhibitor AZD1080-loaded MSNs, termed sAZD1080, reduced the expression of PD-1 in CD8^+^ T cells and promoted the release of perforin from CD8^+^ T cells [131]. Relieving immunosuppressive factors is an effective strategy to boost the immunotherapy efficiency. Thus, combination MNDDSs loaded immunosuppressive factor inhibitor and immunotherapy could realize significantly achievement.

#### Inhibition of the immunosuppressive subpopulation

As one of the main immunosuppressive components in the TME, inhibiting the function of M2 TAMs or depressing the transformation of M2 TAMs into M1 TAMs contributes to preserving the immunosuppressive microenvironment. Jiang et al. reported a biomimetic magnetic nanoparticle Fe_3_O_4_-SASS@PLT loaded with sulfasalazine (SAS) and further coated with a platelet (PLT) membrane [132]. Fe_3_O_4_-SASS@PLT-mediated ferroptosis further upregulated the expression of nuclear factor NF-κB family proteins (Nfkb1 and Nfkb2), which effectively promoted macrophage polarization from the immunosuppressive M2 phenotype to the antitumor M1 phenotype. As NO facilitated macrophage polarization to M1 phenotype [133, 134]. Theivendran’s group constructed S-Nitrosothiol (SNO) modified organosilica nanoparticles with a tetrasulfide-containing to produce intracellular NO. High level of NO leading to mitochondrial dysfunction and disruption of the tricarboxylic acid cycle, resulting in polarization of TAMs to M1 phenotype and delayed tumor growth [135]. Thus, both endogenous and exogenous factors can be used to activate promote the transformation of immunosuppressive conditions.

Elimination or suppression of MDSCs is another feasible strategy to relieve the immunosuppressive microenvironment. Zuo et al. designed a mesoporous silica nanoplatform CeO_2_@MSNs@IR780/Met containing metformin (Met), mitochondrial respiratory inhibitor, IR-780, photosensitizer and CeO_2_, an endogenous H_2_O_2_ consumer. Both CeO_2_ and Met could significantly hinder MDSC tumor infiltration and downregulate tissue-specific MDSC PD-L1 expression. Additionally, CeO_2_@MSNs@IR780/Met activated T cells to transform into CTLs, resulting in an enhanced antitumor immune response [136].

Tregs also have significant immunosuppressive effects, which are characterized by the expression of Foxp3, CD25, and CD4 [137]. Targeting Foxp3 can effectively inhibit the immunosuppression of Tregs. Liu et al. developed an MSNP coated with a lipid bilayer and encapsulated the activated chemotherapeutic drug oxaliplatin (1,2-cyclohexane platinum (II) (DACHPt)) in the pore, forming a DACHPt silicasome. Treatment of a KRAS-derived PDAC model with DACHPt silicasome decreased Treg cell number and function [138].

#### Enhanced antitumor immune factors

Developed MNDDSs enhance antitumor immune factors through four main methods: (1) Inducing cancer cells death via ICD or DAMPs release; (2) Nanovaccines; (3) Cytokine carriers; and (4) Co-stimulation of immune cell activation.

Inducing ICD or DAMP release is becoming a new therapeutic strategy to stimulate immune response generation. Zhang et al. prepared a GSH-responsive ICD nanoamplifier containing diselenide-bridged MONs and ruthenium compound (KP1339), which induces ICD and enhances the antitumor immune response [139]. Wang et al. reported an all-round mesoporous nanocarrier consisting of an upconverting nanoparticle core and a large-pore silica shell (UCMS), photosensitizer molecules, indoleamine-2,3-dioxy-genase (IDO) -derived peptide vaccine Al-9 and PD-L1 inhibitors. Near infrared (NIR) light can activate photosensitizers, induce ICD, and promote effector T-cell infiltration [140].

Vaccination is one of the strategies to boost the antitumor immune response. Mesoporous nanomaterials are often used as adjuvants or delivery carriers to stimulate antigen presenting cells (APC) and enhance antitumor immune response due to their biocompatibility, drug delivery/release ability, and tunability of particle size, morphology, structure and surface function [141]. Yang et al. designed an intelligent nanoreactors constructed from a hybrid silica framework incorporated with Fenton’s reagents (Cu^2+^) and tetrasulfide groups, which respectively trigger the Fenton reaction to produce ROS and antioxidant GSH depletion in the DOX treated cancer cells, leading to oxidative stress and amplified ICD. These nanoreactors are intrinsically immunogenic, exhibiting excellent immune-adjuvant activity for stimulating the maturation of APC, which possessed good synergistic effect with ICB (PD-L1 antibody) and exhibited excellent anticancer performance [142]. Take advantage of MNDDSs as immune adjuvants in situ without cargo loading is a major advance in personalized nanomedicine for clinical transformation.

Nanovaccines have been used to deliver molecular adjuvants to DCs, including toll-like receptor (TLR) agonists and TLR agonists [CpG oligonucleotide and monophosphoryl lipid A (MPLA)] to extend the median survival of tumor-bearing mice [143]. Hu et al. prepared a mesoporous silica nanovaccine loaded with adjuvant CpG modified by the B16-F10 cancer cell membrane, which enhanced DC antigen presentation and T-cell immune activation in the presence of anti-CTLA4 [144]. Wang et al. prepared a black mesoporous titanium dioxide (BMT) multifunctional nanovaccine loaded with L-arginine (LA), forming BMT@LA. BMT@LA combined with PD-L1 antibody (αPD-L1) induced a strong antitumor immune response that effectively killed the primary tumor and further inhibited metastasis [145]. As a kind of tumor-specific antigen, neoantigens constitute ideal cancer vaccine targets and have attracted more attention, but the design of mesoporous nanoplatforms has not yet been reported [146].

Cytokines also participate in immune homeostasis and the inflammatory response in the TME [147]. Kong et al. constructed an A/D/I-dHMLB nanoplatform based on lipid-coated degradable hollow MSNs (dHMLBs) co-encapsulated with all-trans retinoic acid (ATRA), DOX and IL-2. The A/D/I-dHMLB could promote cytokine (IFN-γ and IL-12) secretion, further activate tumor-infiltrating T lymphocytes and NK cells, suppress MDSC infiltration, and reduce IL-10 and TGF-β secretion, which ultimately reshapes the immunosuppressive microenvironment and enhances the antitumor effect [148].

Mesoporous nano-costimulation-based immune cell activation is another popular method to activate tumor immunity. Wang et al. treated bone marrow-derived dendritic cells (BMDCs) with stellate fibrous mesoporous silica nanospheres, which significantly promoted BMDCs proliferation; stimulated IFN-γ, IL-2, IL-4, and IL-10 secretion in lymphocytes; increased the secretion of IgG, IgG1, IgG2a, IgM, and IgA in serum; and enhanced effector memory CD4^+^ T and CD8^+^ T cells in the lymph nodes, spleen and bone marrow of mice [149]. Meanwhile, the combination of Poly(I:C) with stellate fibrous mesoporous silica nanospheres significantly reduced the necessary dosage of Poly(I:C) for antitumor immunity, opening up new opportunities for the clinical application of Poly(I:C) in tumor immunotherapy. Table 1 lists some MNDDSs designed for targeting and remodeling the cellular TME.

**Table 1:** Summary of strategies for targeting and remodeling the cellular TME

Cellular TME	Remodeling strategy	MNDDSs	Guest drug	Application	Tumors	Refs.
Tumor blood vessels	Destroying existing vessels	tHMSN	DOX	Endothelial cells and angiogenesis inhibition	Breast cancer	[116]
		MSN-FTC	Suramin/paclitaxel	Migration inhibition of activated endothelial cells and angiogenesis inhibition	Breast cancer	[117]
	Normalizing tumor vessels	M-MSN_VEGF; siRNA@PEI-PEG-KALA	VEGF-siRNA	VEGF gene silence and angiogenesis inhibition	Ovarian carcinoma	[107]
		NPs@DA	DA	Inhibiting migration of vascular endothelial cell and tubule formation	Breast cancer	[122]
		USMNs-CL	SO/UA	Increased cell apoptosis, downregulated expression of EGFR and VEGFR2 proteins	Hepato-cellular carcinoma	[112]
		ODMSN- AQ4N-LOX	LOX/AQ4N	Downregulated VEGF expression and anti-angiogenesis	Breast cancer	[114]
CAFs	Inhibiting function of CAFs	(Losartan + DOX) @HMPBs	LOS/DOX	Inhibiting the secretion function of CAFs;	Breast cancer	[123]
		Pep-APCDs@Fe/DOX-LOS	LOS/DOX/Fe	Enhanced inhibition of LOS on CAF;	Breast cancer	[124]
Immune cells	Removing the immuno-suppressive factors	DTIC@CMSN + aPD1	DITC	Promoted DITC cytotoxicity; enhanced immuno-therapy efficiency	Melanoma	[130]
		sAZD1080	AZD1080	Suppressed PD-1 expression	Colorectal tumor/lung and pancreas cancer	[131]
	Limiting the release or activation of immunosuppressive factors	Fe_3_O_4_-SAS@PLT	SAS	Immune response triggered by ferroptosis; TAMs repolarize to M1 phenotype	Breast cancer	[132]
		DMON-SNO	SNO/Tetrasulfide	Increased intracellular NO to polarize TAMs to M1 phenotype	Breast cancer	[135]
		CeO_2_@MSNs@IR780/Met	Met/IR780/CeO_2_	Hindered MDSCs tumor infiltration, relieved TME hypoxia; enhanced immune response and PDT	Melanoma	[136]
		DACHPt Silicasome	Pt/DACHPt	Chemotherapy; ICD	Pancreas cancer	[138]
	Enhancing the activity of immune factors	MON@KP1339	KP1339	Amplified KP1339 ICD; boost antitumor immune responses	Breast cancer	[139]
		UCMS@Pep-aPDL1	AL-9/Atezolizumab	NIR Laser-mediated PDT and peptide-augmented immune response and ICB therapy	Lung cancer	[140]
		Cu-DMONs/DOX	Cu^2+^/DOX/Tetrasulfide groups	GSH deletion by Fenton reaction; amplified ICD; stimulated APC maturation	Breast cancer	[142]
		MSN-CpG@CM	CpG	Enhanced accumulation in lymph nodes and immune activation	Melanoma	[144]
		BMT@LA + US + αPD­L1	LA	Enhanced oxidative stress; improved antitumor immuno- therapy	Cervical cancer	[145]
		A/D/I-dHMLB	ATRA/DOX/IL-2	Chemo-immuno-therapy	Melanoma	[148]

## MNDDSs reshape the physicochemical microenvironment

### Ameliorating the hypoxia state of the TME

Currently, there are two ways to overcome tumor tissue hypoxia using MNDDSs: (i) deliver O_2_ directly to tumor tissues, and (ii) Promote in situ O_2_ production of tumor tissues.

#### Delivery of O_2_ to tumor tissues

Red blood cells, hemoglobin and perfluorocarbon vesicles have been used to directly delivery O_2_ to hypoxic TME [150]. Although no directly report on using mesoporous nanomaterials as O_2_ carriers to improve tumor hypoxia, it still provides a possible way to transport O_2_. However, there are still challenges as the O_2_ transporter, including low level O_2_ loaded, oxygen leakage and difficulty in co-delivery with other therapeutic drugs. Thus, more smart strategies need to be explored. Moreover, utilization MNDDSs for in situ oxygen production in the TME is currently a promising strategy.

#### O_2_ supplied by catalysis in situ in the TME

Compared with normal tissues, H_2_O_2_ level is higher in the TME. This feature provides a strategy for tumor treatment. Endogenous H_2_O_2_ can be catalytically decomposed to O_2_ in situ. Oxygen can be stimulated by exogenous or endogenous stimuli to produce more reactive free radicals (including H_2_O_2_, ·OH, singlet oxygen(^1^O_2_), etc.), thus improve the efficiency of oxygen-dependent nano-dynamic therapy. MNDDSs loaded with catalase (CAT) or metal oxide could induce local decomposition of H_2_O_2_ to produce O_2_ in tumor tissues. Liu et al. developed a multiscale hybrid catalytic nanoreactor (catalase@MONs, C@M) by integrating mesoporous organosilica nanoparticles (MONs), and CAT. C@M can catalyze H_2_O_2_ to continuously generate O_2_. Additionally, as an on-demand catalytic nanoreactor, C@M can achieve precise tumor localization and efficient high-intensity focused ultrasound (HIFU) surgery, which is highly desirable for clinical HIFU application [151]. Huang et al. designed a novel hollow mesoporous double-shell Co_9_S_8_@MnO_2_ nanoplatform loaded with the molecular photodynamic agents indocyanine green (ICG) and DOX. The designed MnO_2_ shell nanoplatform can be used as a TME-responsive oxygen self-sufficient producer to alleviate tumor hypoxia and improve photodynamic therapy (PDT) efficiency [152]. Zhang et al. constructed a biodegradable BiPT-PFA nanocomposite by loading platinum (Pt) nanodots into mesoporous bismuth (Bi) nanoparticles. Pt nanodots in the nanocomplex can catalyze the decomposition of H_2_O_2_ to produce O_2_ to alleviate hypoxia, further enhancing the tumor radiation sensitization effect of PFA [153].

In addition to catalyzing the oxygen production of H_2_O_2_ in situ, MNDDSs can also load multivalent metal ions and oxidase to assemble an O_2_ generator to supply O_2_ to the TME. For example, You et al. reported the self-catalyzed Fenton nanosystem (TA/Fe@GOD@DMONs) loaded with natural glucose oxidase (GOD) and tannic acid (TA) grafted using Fe^3+^ on the surface, GOD decomposes glucose to produce H_2_O_2_, and TA accelerates the conversion of Fe^3+^/Fe^2+^, greatly improving the efficiency of Fenton reaction, and catalyzing effective CDT to inhibit tumor [154]. Similar combinations also occur in the combination of oxidase (GOD、LOX) and multivalent metals (such as Mn^2+^、Cu^2+^) [155, 156]. These new ideas represent a new paradigm for the development of autocatalytic O_2_ generated nano-systems for effective treatment.

### Destruction of redox homeostasis

As mentioned in 2.3.3 above, overexpressed GSH could maintain the redox homeostasis by eliminating ROS, which attenuates tumor sensitivity to radiotherapy, chemotherapy and CDT. Three strategies could be explored: (1) expanding ROS generation in the TME; (2) consuming the existing GSH; (3) suppressing the generation of GSH and accelerating its excretion [157].

#### Expanding ROS generation in the TME

The production and elimination of ROS play an important role in maintaining the redox homeostasis of tumor tissues [158]. Although the concentration of ROS is high in cancer cells, it is insufficient to kill cancer cells. Thus, excessive ROS production in tumor tissues is currently a popular strategy for tumor treatment, such as enzyme-catalyzed therapy (ROS-producing enzyme or enzyme complex, peroxidase, glucose oxidase (GOx), etc. Huo et al. prepared mesoporous silica loaded with GOx and Fe_3_O_4_ and formed GOx-Fe_3_O_4_@DMSN. GOx produce a large amount of H_2_O_2_, and ·OH is produced by the Fenton reaction catalyzed by Fe^2+^ to boost cancer cell apoptosis [159]. Shao et al. synthesized IONP-GOD@ART for collaborative therapy using GOD-modified mesoporous iron oxide nanoparticles (IONPs) loaded with artemisinin (ART). In an acidic environment, the nanomaterials gradually decomposed and released Fe^2+^/Fe^3+^, GOD and ART, and GOD and Fe^2+^ formed a “metal oxidase” cascade catalytic system. In addition, unstable endoperoxide bridged in ART were destroyed in the presence of Fe^2+^, producing numerous ROS, which further induced ICD in cancer cells and enhances tumor immunity. IONP-GOD@ART can completely inhibit tumor growth and distant metastasis [160]. Huang et al. successfully prepared mesoporous silica nanoplatform MSNs-PFH@PDA-ICG-PEG-FA loaded with ICG and polydopamine (PDA) layers and coated them with polyethylene glycol-FA. After irradiation by NIR at 808 nm, MSNs-PFH@PDA-ICG-PEG-FA can not only effectively generate heat to achieve photothermal therapy (PTT), but produce ROS to enhance PDT efficiency [161].

#### Consuming the existing GSH

GSH-depleted anticancer nanodrugs could promote the effect of ROS-based tumor therapeutic efficacy [162]. Lin et al. developed a versatile and bacteria-like PEG/Ce-Bi@DMSN nanozyme by coating Bi_2_S_3_ nanorods (NRs) with dendritic mesoporous silica (Bi_2_S_3_@DMSN) and then decorating Bi_2_S_3_@DMSN with ultrasmall ceria nanozymes. The nanozymes showed dual enzyme-mimic catalytic activities (peroxidase-mimic and CAT-mimic) under acidic conditions, and effectively consume overexpressed GSH through redox reactions, which simultaneously elevate oxidative stress and alleviate hypoxia and significantly improving ROS-mediated therapeutic efficiency [163]. Simultaneously depleting GSH and increasing ROS represents a promising avenue [164]. Hu et al. combined FA-modified mesoporous dopamine nanoparticles (FA-MPPD) with new indocyanine green (IR-820) and perfluorooctane (PFO) to form the nanoplatform IR-820/PFO@FA-MPPD, which integrates the functions of ROS supply, GSH consumption and tumor targeting, ultimately enhancing the PDT tumor inhibition effect [165].

**Fig. 5:**
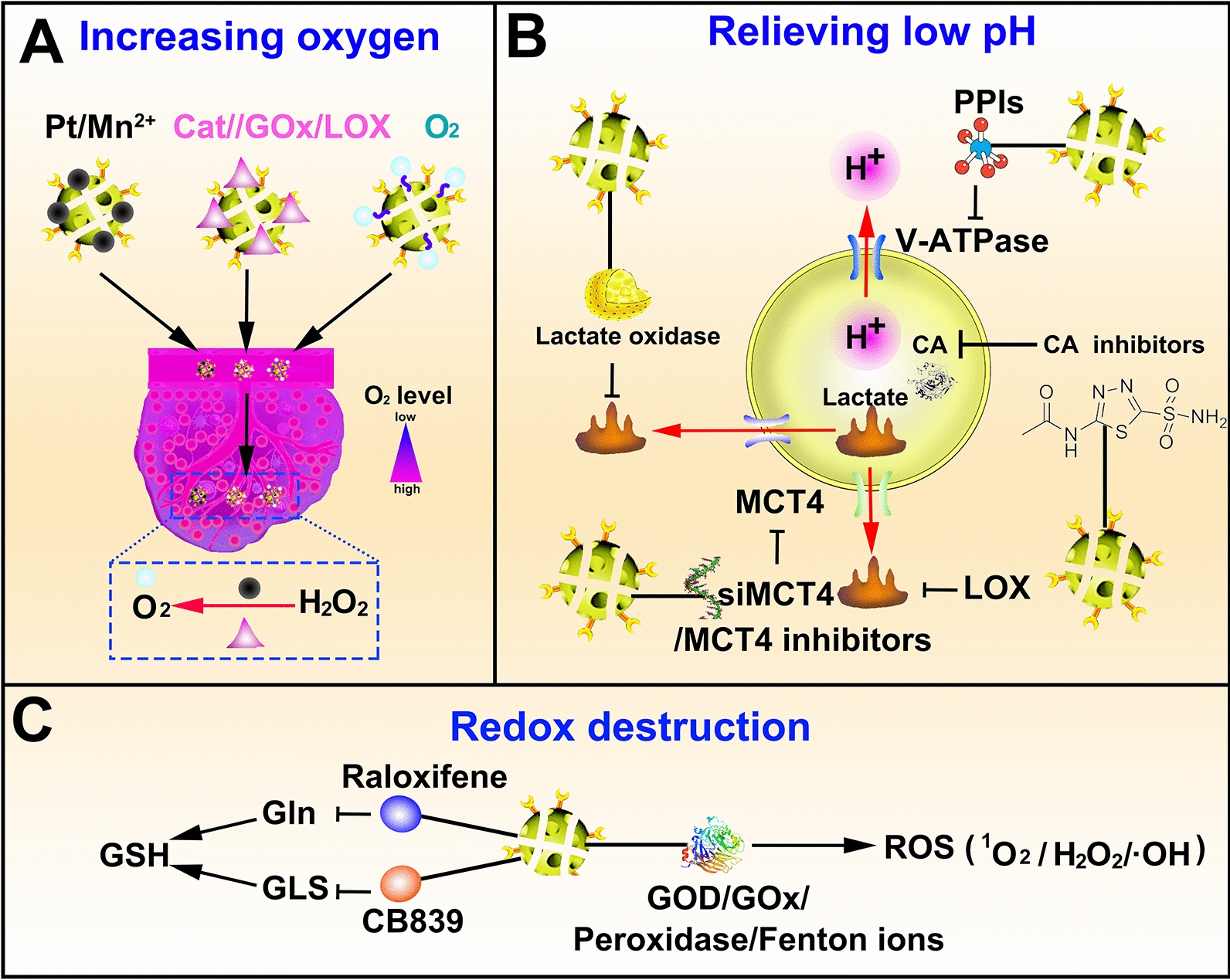
Strategies for reshaping the physicochemical microenvironment. **A** Ameliorate the hypoxia state of the TME in the methods of delivering O_2_ or catalyzing O_2_ production in situ of TME. **B** Relieve the low pH state of the TME by blocking the efflux of intracellular acid metabolites or promote intracellular acid metabolites generation to induce cancer cells acidosis. **C** Destruct redox homeostasis in the forms of increased ROS generation in the TME, existing GSH consumption and attenuated GSH generation

Taking advantage of sulfide and high-valence metal ions could also diminish the intracellular GSH level. Dendritic mesoporous organosilica nanoparticles (GDMONs) with a tetrasulfide-incorporated framework reported by Yu et al. could decrease the intracellular GSH level through -S‒S-/GSH redox chemistry, increase ROS production in vitro and in vivo, facilitate CTLs proliferation, and reduce the growth of aggressive melanoma models [166]. Moreover, Lin et al. synthesized multifunctional dendritic mesoporous organosilica (DMOS) co-incorporated with manganese ions, iron ions or cobalt ions and tetrasulfide bonds to deliver ICG. In the TME, hydrogen sulfide (H_2_S) produced by the reaction between tetrasulfide bonds and overexpressed GSH results in mitochondrial injury to reduce cellular respiration. Additionally, the released Mn^2+^ catalyzes endogenous H_2_O_2_ to produce O_2_. Both GSH depletion and trimodal O_2_ compensation significantly improve the PDT efficiency of ICG [167]. Ma’s group synthesized bimetallic Zn^2+^/Cu^2+^ co-doped hollow mesoporous organosilica (HMOS@MOF), used for targeted delivery of cisplatin (cis-diaminodichloro platinum (CDDP). Cu^2+^ can consume intracellular GSH and catalyze the decomposition of H_2_O_2_ into highly toxic • OH. Seriously reduced GSH could protect the • OH from scavenging, greatly improving the CDT effect of • OH group and the toxicity of CDDP [168]. As a widely studied strategy to destroy the TME homeostasis, GSH depletion plays a significant role in synergizing tumor radiotherapy and chemotherapy (Fig. 5).

#### Suppressing the generation of GSH and accelerating its excretion

Glutamate cysteine ligase (GCL) and glutathione synthetase and glutaminase (GLS) is glutathione synthesis rate-limiting enzyme. Glutamic acid, cysteine and glycine are GSH synthesis raw materials. Blocking either rate-limiting enzyme or raw material would affect the GSH synthesis. Raloxifene could inhibit the intake of glutamine (Gln) and intercept GSH synthesis. Liu et al. prepared mesoporous carbon nanospheres loaded with raloxifene and 2,2-azobis[2-(2-imidazolin-2-yl) propane] dihydrochloride (AIBI). AIBI was decomposed into alkyl radicals to kill cancer cells, while raloxifene inhibited the synthesis of GSH and synergistically enhanced PDT [169]. CB839, a depressor of GLS, was grafted on the bifunctional nanozyme of nano-sized Au and Fe_3_O_4_ coloaded dendritic MSNs (DMSN-Au-Fe_3_O_4_). The nanozyme with Au-mediated H_2_O_2_ self-supply, Fe_3_O_4_-triggered Fenton-like reaction and CB839-mediated GSH depletion significantly boosted the efficacy of CDT, and achieved significant anti-tumor properties in vitro and in vivo [170].

Deletion GSH is an alternative strategy. Consumption of NADPH can inhibit the reduction of GSSG to GSH, thereby decreasing the production of GSH. The efflux of GSSG is closely related to the multidrug resistance associated protein-1 (MRP-1) efflux pump [171]. Therefore, regulating MRP-1 could accelerate exporting GSH, thus enhance the oxidative stress of TME and improve the cancer cells therapeutic resistance. The related clues in the field of mesoporous nanomaterials research just in infancy, while it provides us with some new strategies to reduce intracellular GSH.

### Relieve the low pH state of the TME

Acid metabolites molecules (H^+^, lactate, carbonic acid, etc.) construct low pH values that may result in cancer occurrence and development. Therefore, deleting acid molecules or relieving low pH conditions is a promising cancer treatment. Currently, the common ways to relieve the low pH value include downregulating intracellular and extracellular acid substances, blocking the exportation of intracellular acid substances, and consuming intracellular and extracellular acid substances.

Carbonic anhydrase (CA) inhibitors (CAIs) have been developed to reduce carbonic acid levels, which indirectly reduce the acidity of the TME [172, 173]. Chen et al. modified MSNs with an anti-CAIX antibody (A-CAIX Ab) and DOX via disulfide bonds and developed a new antibody-targeting and GSH-responsive nanocomposite particle, DOX@MSNS-CAIX. DOX@MSNS-CAIX could accumulate in tumors, relieve the low pH, and induce more cancer cell apoptosis in 4T1-bearing mice [174].

As a special exporter of lactate, MCT inhibition is an alternative method to block acid substances. Li et al. constructed a hollow mesoporous organosilica nanoparticle loaded with hydroxycamptothecin (HCPT) and monocarboxylate transporter 4 (MCT4) interfering RNA (siMCT4), which inhibits the efflux of lactate from cancer cells. Moreover, the reduction in extracellular lactate can promote the transformation of TAMs from the M2 type to the M1 type, restore the activity of CD8^+^ T cells in vivo, alleviate the immunosuppression of the microenvironment, and effectively inhibit the proliferation of B16F10 tumors and lung metastasis of 4T1 cells [175]. Moreover, proton pump inhibitors (PPIs) and Na^+^/H^+^ exchange inhibitors are known to be involved in pH regulation and contribute to relieving the acidic TME [176–178]. Regrettably, there are currently no reports on the design of mesoporous nanoplatforms based on these drugs.

Consumption of the acid metabolites also assists in alleviating the low pH of the microenvironment. Tang et al. reported that dendritic MSNs loaded with lactate oxidase could increase the consumption of lactate in the TME. Lactate consumption downregulates VEGF expression and resists tumor angiogenesis and metastasis. Moreover, consumption of lactate catalyzed by LOX produces cytotoxic H_2_O_2_, which leads to oxidative damage and increased hypoxia levels to enhance antitumor and antimetastatic efficacy [114]. Chen et al. incorporated Met and fluvastatin sodium (Flu), an MCT4 inhibitor, into MnO_2_-coated MSNs to construct the tumor-targeting nanoplatform Me&Flu@MSN@MnO_2_-FA. Met can promote the production of more lactate by cancer cells, while Flu inhibits the efflux of lactate, which leads to an acidosis intracellular microenvironment and cancer cell death. Due to the limited efflux of lactate, the extracellular lactate concentration is reduced, and the migration ability of cancer cells is also weakened [179].

## Reshaping the biological microenvironment

### Remodeling energy metabolism

Energy metabolites play an indispensable role in maintaining biological tumor behavior. At present, MNDDSs-based TME energy metabolism remodeling focuses mainly on blocking or consuming energy metabolism materials and inhibiting the Warburg effect and reverse Warburg effect.

#### Blocking or consuming energy metabolism raw materials

**Fig. 6:**
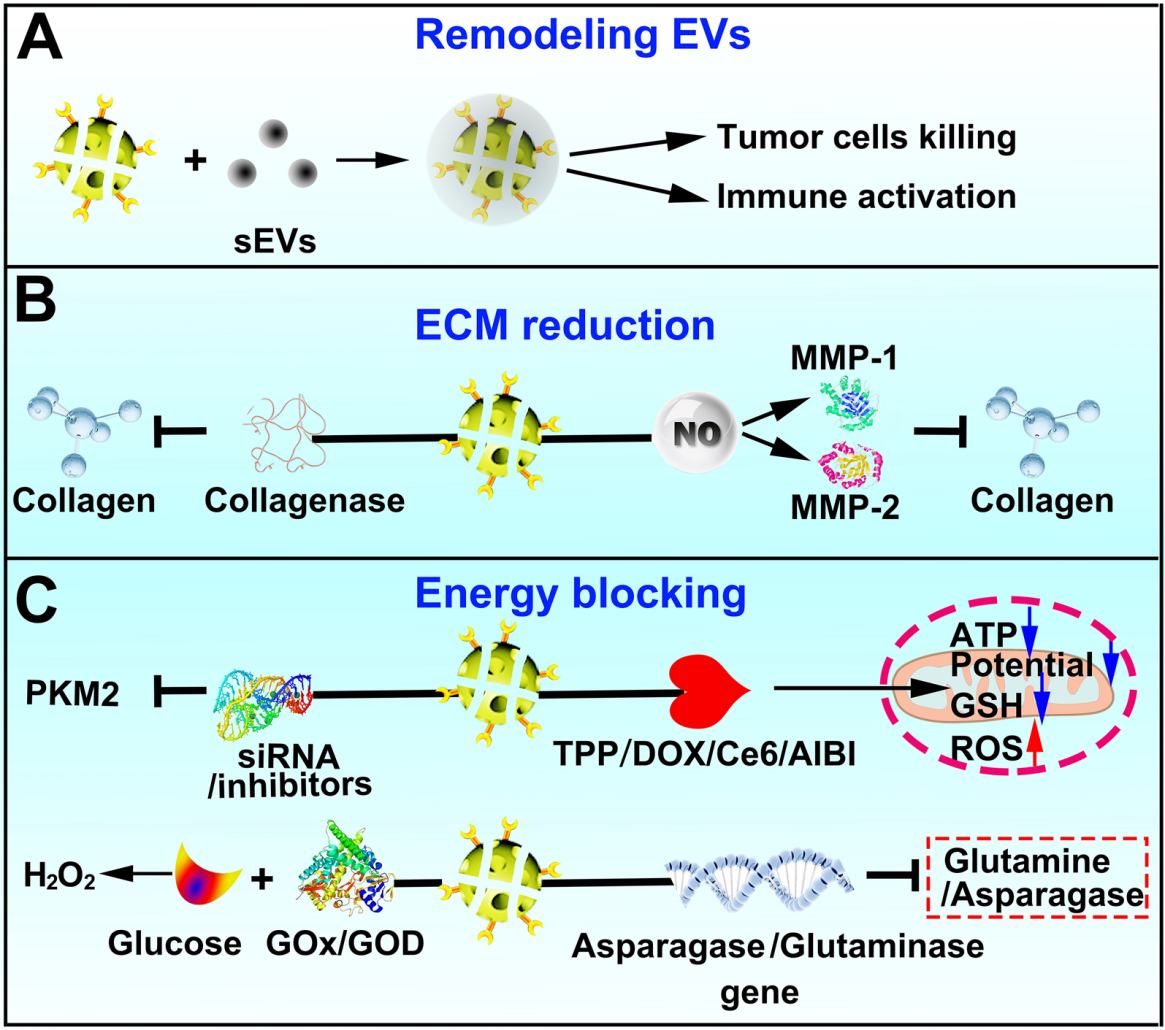
Strategies for reshaping the biological microenvironment. **A** Remove tumor-derived sEVs from circulation to increased intestinal A-Exo, and attenuated A-Exo-induced tumor metastasis. **B** Regulate collagen degradation and decomposition to reshape the ECM. **C** Block or consume energy metabolism raw materials (such as glucose, lactate, glutamine, etc.) or silence gene of energy metabolism to induce cancer cell death

Destroying tumor blood vessels is a traditional avenue to block energy materials. Blocking or consuming energy metabolism materials (such as glucose, lactate, glutamine, etc.) in the TME is a novel strategy for starvation cancer therapy (Fig. 6). GO_X_, which catalyzes the oxidation of glucose to produce H_2_O_2_ and gluconic acid, consumes a large amount of glucose and O_2_, which could significantly enhance synergistic chemotherapy, phototherapy, and immunotherapy [180]. Mesoporous nanomaterials loaded with GO_X_, peroxidase, prodrugs, polyvalent metal ions and other substances may realize multimodal cancer combination therapies. Shan et al. designed organosilica-based hollow mesoporous bilirubin nanoparticles (HMBRNs) coloaded with GOx and tirapazamine (TPZ), which rapidly depleted glucose and oxygen in tumors and enhanced starvation therapy and chemotherapy, with fewer side effects [181]. Huo et al. integrated GOD and ultrasmall Fe_3_O_4_ nanoparticles into large pore-sized, biodegradable dendritic silica nanoparticles to prepare a sequential nanocatalyst. GOD in nanocatalysts can effectively deplete glucose and generate a large amount of H_2_O_2_ to catalyze Fe_3_O_4_ through Fenton-like reactions, which ultimately trigger cancer cell death [159].

#### Inhibiting or reversing the Warburg effect

Cancer cells and stromal cells rely mainly on the “Warburg effect” to acquire energy materials. Recently, the “reverse Warburg effect” was also revealed to be involved in cancer cell biogenesis [182]. Thus, effectively delivering these enzyme inhibitors or siRNA may block the Warburg effect or reverse the Warburg effect, representing a promising tumor therapy. Shen et al. developed a universal siRNA vector consisting of cyclodextrin-grafted polyvinyl imine (CP)-functionalized MSNPs. In vitro and in vivo experiments have shown that CP-MSCs can effectively inhibit pyruvate kinase 2 (PKM2) gene expression, further inhibiting cancer cell growth, invasion and migration [183]. In a later report, Shen et al. designed a highly efficient mesoporous silica nanoplatform, CP-MSNP@DOX/siRNA, for the codelivery of DOX and PKM2 siRNA oligomers, which showed a good effect of the combination of gene and chemotherapy in a mouse triple-negative breast cancer model [184].

### Reduce the generation of ECM

Downregulating the expression of ECM or degrading the generated ECM will help improve the delivery efficiency in tumor tissues. As collagen is the main component of the ECM, regulating collagen degradation and decomposition has become a common target for reshaping the ECM. Villegas et al. designed polymerized nanocapsules with hybrid collagenase on the surface of MSNs to improve the infiltration of nanoparticles into tumor tissues depending on hybrid collagenase degradation in a high-density matrix [185]. Besides, exogenous enzymes have often been used to degrade collagen. Based on activating endogenous matrix metalloproteinases (MMPs-1 and MMPs-2) using NO, Dong et al. presented MSN loaded with DOX and a NO donor (S-nitrosothiol). Construction of DN@MSN make tumor being more permeable to the nanovehicle and DOX, significantly promoting antitumor efficacy with less toxicity [186]. However, no administration of MNDDSs have been reported in collagen denaturation, which thought to be a promising strategy [187].

As another important component of the ECM, reduced HA can loosen the ECM skeleton, promote antitumor drug infiltration and relieve high interstitial pressure. Delivering hyaluronidase or HA inhibitors using mesoporous nanomaterials enhanced antitumor drug penetration into tumor tissue. CD44 can bind specifically to HA and its derivatives to achieve targeted drug delivery through a receptor-ligand mechanism [188, 189]. Fang et al. synthesized HA-modified MSNs (HA-MSNs) coated with superparamagnetic Fe_3_O_4_, which realized high tumor penetration [190].

### Remodeling extracellular vesicles

Tumor-derived small extracellular vesicles (sEVs) play a critical role in regulating the TME and further tumor progression and metastasis. Explicitly removing tumor-derived sEVs from circulation has been proposed. Xie et al. used positively charged MSNs functionalized with EGFR-targeting aptamers (MSN-AP) to specifically recognize and bind blood-borne negatively charged oncogenic exosomes (A-Exo) and deliver A-Exo through the hepatobiliary layer and Oddi's sphincter into the small intestine, which significantly decreased circulating A-Exo levels, increased intestinal A-Exo, and attenuated A-Exo-induced lung metastasis in mice [191].

## Conclusion and outlooks

The persistence and complexity of the TME results in tumors being more aggressive. The components of the TME, such as hypoxia, nutrient deficiency, weak acidity, high ROS and GSH, an immunosuppressive microenvironment, and a viscous ECM, are closely related to cancer cell survival, proliferation, metastasis, and treatment resistance. Additionally, cancer cells can further deteriorate the TME through metabolic reprogramming, forming a vicious cycle. Combining cancer cells and TME therapy represents a promising strategy.

MNDDSs have excellent physical and chemical properties, which can safely, efficiently and accurately deliver agents to tumor tissues, specifically in blocking the interaction between the TME and cancer cells, and ultimately achieve the purpose of direct treatment or synergistic sensitization treatment of tumors. Currently, MNDDSs-based targeted remodeling TME including: (1) remodeling cellular TME, involved decreasing or normalizing tumor vessels, regulating functions of CAFs and remodeling immune microenvironment; (2) reshaping the physicochemical microenvironment, involved improving oxygen supply, destructing redox and pH homeostasis; (3) reshaping the biological microenvironment, involved remodeling energy metabolism, reducing ECM generation, and improving function of extracellular vesicles. Table 2 summarizes some strategies MNDDSs applied for targeting and remodeling the biophysiochemical TME.

**Table 2:** Summary of strategies for targeting and remodeling the biophysiochemical TME

Biophysiochemical TME	Remodeling strategy		Nano-carrier	Guest drug	Application	Tumor types	Refs.
Physicochemical TME	Destroying REDOX Homeostasis	Catalyzing H_2_O_2_ in situ to produce O_2_	Co_9_S_8_@MnO_2_-ICG/DOX	ICG/DOX	Self-generated oxygen enhanced PDT; promoted chemotherapy	Ovarian adenocarcinoma	[152]
			BiPt-PFA	Pt/Bi dots	Alleviated hypoxia; GSH depleted; sensitization radiation	Breast cancer	[153]
		Oxidase delivered to decompose metabolites and produce O_2_	TA/Fe@GOD@DMONs	TA/Fe/GOD	Decomposing GOD to glucose and producing H_2_O_2_; effective CDT	Breast cancer	[154]
		Expanding ROS generation in the TME	GOD-Fe_3_O_4_@DMSN	GOD/ultrasmall Fe_3_O_4_ NPs	Glucose exhausted and Fenton reaction produced ROS to boost cancer cell apoptosis	Breast cancer	[159]
			IONP-GOD@ART	GOD/ART/Fe	Numerous ROS produced by "Metal oxidases" cascade and induce ICD	Breast cancer	[160]
			MSNs-PFH@PDA-ICG-PEG-FA	PFH/ICG	Self-generated ROS enhanced PDT	Breast cancer	[161]
		Consuming the existing GSH	PEG/Ce-Bi@DMSN	Ultrasmall ceria nanozymes	Elevated oxidative stress and relieved hypoxia in the TME	Cervical cancer	[163]
			IR-820/PFO@FA-MPPD	IR-820/PFO	Hypoxia relieved; GSH depletion; enhanced PDT effect	Hepatocellular carcinoma	[165]
			GDMON-P + OVA + CpG	Ovalbumin/a TLR9 agonist	A self-adjuvant for cancer vaccine; deplete GSH; increased ROS	Melanoma	[166]
			Mn-Fe- Co DMOS NPs	ICG	GSH depletion and improved O_2_-depended PDT	Breast cancer	[167]
			HMOS@MOF	CDDP/Zn^2+^/Cu^2+^	GSH depletion; enhanced CDT and chemotherapy	NSCLC	[168]
		Suppressing the generation of GSH	MCN-AR@PCM	AIBI/Raloxifene	GSH synthesis suppression and O_2_-irrelevant radicals produced by AIBI to kill cancer cells	Breast cancer/glioblastoma	[169]
			DMSN-Au-Fe_3_O_4_	Au/Fe_3_O_4_	Au-mediated H_2_O_2_ self-supply and GSH biosynthesis inhibition	Breast cancer	[170]
	Reverse low pH of TME	Inhibiting the outflow of intracellular acid metabolites	DOX@MSNs-CAIX	DOX/A‑CAIX Ab	CAIs delivered to reduce TME Carbonic acid levels; Promoted toxicity of DOX	Breast cancer	[174]
			HMONs@HCPT-BSA-PEI-CDM-PEG@ siMCT-4	HCPT/siMCT-4	Lactate efflux inhibition by role of siMCT4; enhanced chemotherapy	Melanoma/breast cancer	[175]
		Consumption of extracellular acidic metabolites	ODMSN-AQ4N-LOX	LOX/AQ4N	Lactate depletion metastasis and angiogenesis resistance; increased tumor hypoxia	Breast cancer	[114]
		Promoting intracellular acidic metabolites production and inhibit their effluvia	Me&Flu@MSN@MnO_2_-FA	Me/Flu	Increased intracellular lactate and decreased extracellular lactate; cancer cells acidosis	Breast cancer	[179]
Biological TME	Remodeling energy metabolism	Consuming energy metabolites	HMBRN-GOx/TPZ	Gox/TPZ	Glucose consumption to starve cancer cells and increased TPZ toxicity	Astroblastoma/Breast cancer	[181]
		Inhibiting Warburg effect	CP-MSNP@DOX/siRNA	DOX/PKM2 siRNA	PKM2 gene silence to inhibit Warburg effect; enhanced chemotherapy	Breast cancer	[184]
	Remodeling ECM	Reduce ECM	MSN-Col-nc	Collagenase	Extracellular matrix degradation	Osteosarcoma	[185]
			DN@MSN	DOX/a NO donor (S-nitrosothiol)	MMPs activated by NO to degrade collagen in the ECM	Breast cancer	[186]
	Remodeling extracellular vesicles	Decrease circulating sEVs levels	MSN-Exo	A-Exo	Decreased circulating A-Exo levels; attenuate A-Exo-induced lung metastasis	Lung cancer	[191]

To date, mesoporous oxides (SiO_2_, MnO_2_, Fe_3_O_4_), mesoporous platinum, mesoporous carbon, mesoporous nitride, phosphate, and sulfide have been designed for tumor target therapy. However, there are still some challenges that limit treatment efficiency and clinical transformation. Firstly, MNDDSs biosafety. The widely studied MSNs have stable inorganic rigid skeleton with slow degradation rate, which can remain in the body for several weeks to several months. In order to solve this dilemma, various methods have been developed to accelerate the degradation of MSNs, such as introducing organic parts/metal ions into the Si-O-Si skeleton of MSN, bridging MSN with selenium/disulfide. Therefore, MNDDSs with skeletal responsive biodegradation characteristics should be further developed to achieve biocompatibility and ensure the effectiveness of free radicals in nano-dynamic therapy. Secondly, drug loading and functional modification. Although the strong drug loading and functional modification ability, there are still several limitation and problems, such as small drug loading, drug leakage, tumor treatment residue, single treatment resistance, and side effects on surrounding tissues. Further tune the physical and chemical characteristics of mesoporous materials may contribute to solve this problem. Hollow mesoporous materials and sandwich mesoporous materials not only provide more space, but also prevent the premature release and degradation of drugs, regulate the therapeutic agents release, reduce side effects, and ultimately may improve the results of anti-cancer treatment. Dendritic mesoporous materials are also designed to connect multiple functional modification groups, reduce drug leakage, and better respond to the endogenous stimulation of the TME. Finally, in terms of internal circulation and action efficiency, most of the current nano materials displayed poor stability and low energy conversion efficiency. Designed MNDDSs wrapped in red cell membrane or cancer cell membrane may avoid to be swallowed by immune cells, and increase biocompatibility and circulation time. The latter is beneficial to the homologous uptake of cancer cells and the activation of immunity in situ as artificial antigen. Flexible organic mesoporous materials, with long cycle time in vivo and high cell uptake efficiency, have currently become attractive candidates. Many responsive substances have been used to modify MNDDSs for stimulating responsive drug cascade release and stimulus-responded nano-dynamic therapy, significantly enhancing the local therapeutic effect of the lesion. How to optimize the internal circulation and tissue uptake of MNDDSs is worthy of exploration, which is of great significance for clinical transformation.

In addition, MNDDSs still face some challenges in reshaping TME. MNDDSs with excellent targeting performance, high specific tumor uptake rate and multi-level drug delivery at different stages have yet to be designed. How to better control the time and space transfer of in vitro drug delivery platform is a challenge and needs further research. The design of a high biosafety, comprehensive and stable mesoporous nano-drug delivery system to optimize cancer treatment, and the in-depth study of the interaction and regulatory mechanism between tumor and microenvironment will accelerate the clinical transformation process and show a better prospect for the collaborative and efficient treatment of cancer.

## Data Availability

Not applicable.

## Abbreviations

AIBI: 2,2-Azobis[2-(2-imidazolin-2-yl) propane] dihydrochloride
APC: Antigen presenting cells
ART: Artemisinin
BMDCs: Bone marrow-derived dendritic cells
CAFs: Cancer-associated fibroblasts
CAIs: Carbonic anhydrase inhibitors
CAT: Catalase
CCL2: Chemokine ligand 2
CDDP: Cis-diaminodichloro platinum
CDT: Chemodynamic therapy
CSCs: Cancer stem cells
CTLA-4: Cytotoxic T-lymphocyte-associated protein 4
CTLs: Cytotoxic T cells
CXCL9: C–X–C motif chemokine ligand 9
DA: Dopamine
DOX: Doxorubicin
DAMPs: Damage-associated molecular patterns or antigen
DCs: Dendritic cells
ECM: Extracellular matrix
EGFR: Epidermal growth factor receptor
EPR: Enhanced permeability and retention
EVs: Extracellular vehicles
FA: Folic acid
FAP: Fibroblast activation protein
Flu: Fluvastatin sodium
GAS6: Growth arrest specific protein 6
GOx (GOD): Glucose oxidase
GSH: Glutathione
HA: Hyaluronic acid
HGF: Hepatocyte growth factor
HCPT: Hydroxycamptothecin
HIF-1α: Hypoxia inducible factor 1-alpha
HIFU: High-intensity focused ultrasound
ICD: Immunogenic cell death
ICG: Indocyanine green
IDO: Indoleamine-2,3-dioxy-genase
IFN: Interferon
IL: Interleukin
LAG-3: Lymphocyte activation gene-3
KLF2: Kruppel-like factor-2
LOS: Losartan
LOX: Lactate oxidase
MAPK: Mitogen-activated protein kinase
MCT4: Monocarboxylate transporter 4
MDR: Multiple drug resistance
MDSC: Myeloid-derived suppressor cell
Met: Metformin
MMPs: Matrix metalloproteinases
MNDDSs: Mesoporous nanodrug delivery systems
MPLA: Monophosphoryl lipid A
MRP-1: Multidrug resistance associated protein-1
MSN: Mesoporous silica nanoparticle
NADPH: Niacinamide adenine dinucleotide phosphate
NIR: Near infrared
NK: Natural killer
NO: Nitric oxide
NSCLC: Non-small cell lung cancer
OXPHOS: Oxidative phosphorylation
PD-1: Programmed cell death 1
PDA: Polydopamine
PDAC: Pancreatic ductal adenocarcinoma
PDT: Photodynamic therapy
PI3K: Phosphatidylinositol-3-OH kinase
PKM2: Pyruvate kinase 2
PPIs: Proton pump inhibitors
PTT: Photothermal therapy
ROS: Reactive oxygen species
RTK: Receptor tyrosine kinase
SAS: Sulfasalazine
siMCT4: Monocarboxylate transporter 4 (MCT4) interfering RNA
sEVs: Small extracellular vehicles
SO: Sorafenib
TAECs: Tumor-associated endothelial cells
TAMs: Tumor-associated macrophages
TIM-3: T-cell immunoglobulin-3
TKIs: Tyrosine kinase inhibitors
TLR; toll-like receptor; TME: Tumor microenvironment
TPZ: Tirapazamine
UA: Ursolic acid
VEGF: Vascular endothelial growth factor
VEGFR2: Vascular endothelial growth factor receptor 2
α-SMA: α-Smooth muscle actin
